# Metronidazole conjugated magnetic nanoparticles loaded with amphotericin B exhibited potent effects against pathogenic *Acanthamoeba castellanii* belonging to the T4 genotype

**DOI:** 10.1186/s13568-020-01061-z

**Published:** 2020-07-17

**Authors:** Sumayah Abdelnasir, Ayaz Anwar, Muhammad Kawish, Areeba Anwar, Muhammad Raza Shah, Ruqaiyyah Siddiqui, Naveed Ahmed Khan

**Affiliations:** 1 Department of Biological Sciences, School of Science and Technology, Sunway University, Subang Jaya, 47500 Selangor, Malaysia; 2 HEJ Research Institute of Chemistry, International Center for Chemical and Biological Sciences, University of Karachi, Karachi, 75270 Pakistan; 3 Faculty of Defence Science and Technology, National Defence University of Malaysia, 57000 Kuala Lumpur, Malaysia; 4 Department of Biology, Chemistry and Environmental Sciences, College of Arts and Sciences, American University of Sharjah, Sharjah, 26666 United Arab Emirates

**Keywords:** Magnetic nanoparticles, Metronidazole, Amphotericin B, *Acanthamoeba*

## Abstract

*Acanthamoeba castellanii* can cause granulomatous amoebic encephalitis and *Acanthamoeba* keratitis. Currently, no single drug has been developed to effectively treat infections caused by *Acanthamoeba*. Recent studies have shown that drugs conjugated with nanoparticles exhibit potent in vitro antiamoebic activity against pathogenic free-living amoebae. In this study, we have developed a nano drug delivery system based on iron oxide nanoparticles conjugated with metronidazole which were further loaded with amphotericin B to produce enhanced antiamoebic effects against *Acanthamoeba castellanii*. The results showed that metronidazole-nanoparticles-amphotericin B (Met-MNPs-Amp) significantly inhibited the viability of these amoebae as compared to the respective controls including drugs and nanoparticles alone. Met-MNPs-Amp exhibited IC_50_ at 50 μg/mL against both *A. castellanii* trophozoites and cysts. Furthermore, these nanoparticles did not affect the viability of rat and human cells and showed safe hemolytic activity. Hence, the results obtained in this study have potential utility in drug development against infections caused by *Acanthamoeba castellanii*. A combination of drugs can lead to successful prognosis against these largely neglected infections. Future studies will determine the value of conjugating molecules with diagnostic and therapeutic potential to provide theranostic approaches against these serious infections.

## Introduction

Nanoparticles (NPs) are proven future antimicrobials against bacteria, fungi, viruses, and parasites because of their broad activities against microbes including but not limited to the production of reactive oxygen species, DNA interaction, and metabolic functions (Hoseinzadeh et al. 2017; Vimbela et al. 2017; Wang et al. 2017). These characteristics of metallic nanoparticles improve the capacity of drug development against infectious diseases. Their physical properties including, size, surface charge, morphology and structure play pivotal roles in governing their antimicrobial potential (Ahmed et al. 2017; Anwar et al. 2019a, b, c). Metallic oxide nanoparticles including ZnO, CuO, Fe_2_O_3_, NiO and MgO etc. have shown potential in antimicrobial applications due to their easily modified surface functionalization with peptides, antibodies and therapeutic agents (Raghunath and Perumal 2017). Among metal oxide NPs, iron oxide NPs have been used most widely against various pathogenic bacteria due to potential bactericidal effects owing to their smaller size, magnetism, photothermal property and biocompatibility (Azam et al. 2012). However, to date, there are very few reports that account the affinity of any metal oxide nanoparticles against amoebae. In two recent reports, TiO_2_ nanoparticles have shown some potential against *Acanthamoeba castellani* triggered by external stimuli in both cases (Gomart et al. 2018; Imran et al. 2016a, b). Another report showed the limited efficacy of cobalt oxide (Co_3_O_4_)NPs as compared to cobalt phosphate (Co_3_(PO_4_)_2_) NPs against *A. castellanii* (Anwar et al. 2019a). Nanoparticle conjugation with broad spectrum antibiotics and natural products, however, has been found to be an effective strategy against brain-eating amoebae, particularly, *N. fowleri* (Anwar et al. 2019b, c; Rajendran et al. 2017).

Pathogenic free-living amoebae, including *Naegleria fowleri*, *Balamuthia mandrillaris*, and *Acanthamoeba castellanii* are opportunistic protists that cause infection of the central nervous system (CNS) (Visvesvara 2013; Schuster and Visvesvara 2004). Infection of the CNS by free-living amoebae almost always proves to be fatal (Visvesvara et al. 2007). This indicates the virulent nature of the pathogenic amoebae and the lack of effective treatment options against these parasites. The lack of awareness, lack of diagnostic modalities and lack of public knowledge about amoebae are considered to be one of many reasons of widespread amoebic infections worldwide (Ali et al. 2020). In addition, amoebae-related CNS infections are often misdiagnosed as other CNS infections, such as bacterial meningitis, due to the similarity in symptoms (Siddiqui and Khan 2014).

The blood–brain barrier (BBB) typically hampers the delivery of drugs into the CNS where the parasites usually reside. As a result, a higher dosage of drugs is required to treat this infection, which contributes to the severe side effects. Patients suffering from granulomatous amoebic encephalitis (GAE) are usually treated with multi-drug regimens which often include amphotericin B, miltefosine and pentamidine isethionate but the prognosis of disease remains poor (Cope 2013).

The current study was aimed to develop metronidazole modified iron oxide nanoparticles loaded with amphotericin B (Met-MNPs-AmpB) and to test their antiamoebic potential against *Acanthamoeba castellanii*. We used AmpB as it shows limited effects against *A. castellanii* and the purpose of this study was to determine whether such a formulation would enhance its effects. The developed nanoparticles (Met-MNPs-AmpB) were thoroughly characterized through the determination of size, zeta potential, surface functional groups, and surface morphology using dynamic light scattering (DLS), Fourier transform infrared (FT-IR) spectroscopy and atomic force microscopy (AFM), respectively. The biocompatibility of Met-MNPs-AmpB was evaluated on human cervical cancer cell lines (HeLa), as well as mouse embryonic cell lines (3T3 NIH) using 3-(4,5-dimethylthiazol-2-yl)-2,5-diphenyltetrazolium bromide (MTT) assay. Moreover, hemocompatibility analysis was also performed against fresh human erythrocytes. The antiamoebic properties of these nanoparticles were established against both trophozoite and cyst stages of *A. castellanii* in amoebicidal and cysticidal assays.

## Materials and methods

### Chemicals

All organic solvents utilized in experiments were of high-performance liquid chromatography (HPLC) grade and purchased form Fisher scientific UK. Ferric Sulphatehexahydrate Fe_2_(SO_4_)_3_·6H_2_O, Ferrous sulphateheptahydrate (FeSO_4_·7H_2_O), 3-(Trimethoxysilyl)propyl methacrylate (MPTES), 4-dimethyl amino pyridine (DMAP), azobisisobutyronitrile (AIBN), ammonium hydroxide, dicyclohexylcarbodiimide (DCC), metronidazole and amphotericin B were obtained from Sigma-Aldrich, Merck Darmstadt (Germany) through a local supplier.

### Synthesis of Met-MNPs and Met-MNPs-Amp

**Fig. 1:**
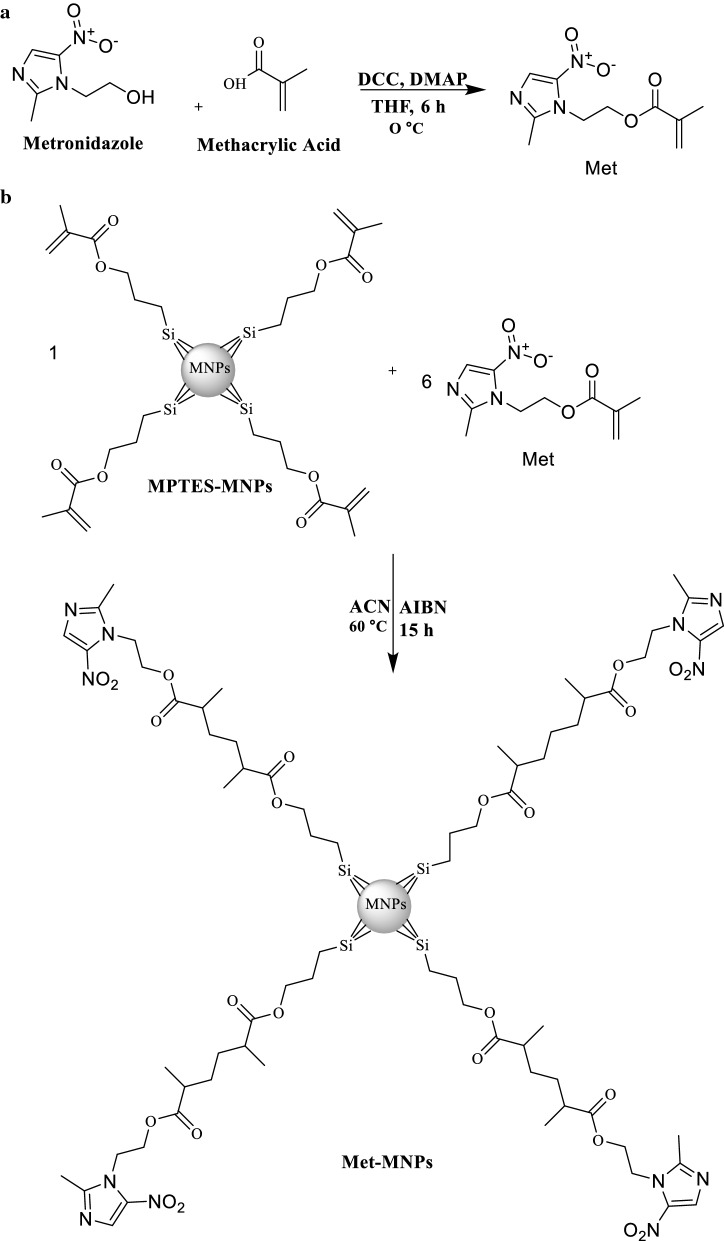
**a** Synthetic scheme of derivative of metronidazole as Met. **b** Met-MNPs

The synthesis of Met-MNPs-Ampwas carried out in multiple steps described in Fig. 1. First, compound 1 was synthesized by the following procedure. Methacrylic acid (0.77 g, 9.0 mmol), 4-Dimethyl amino pyridine (0.061 g, 0.5 mmol) and dicyclohexylcarbodiimide (DCC, 1.85 g, 9.0 mmol) were taken in a round bottom flask containing tetrahydrofuran (THF) (20 mL) connected with a condenser. The reaction was stirred for 10 min at 0 °C under Ar atmosphere. Metronidazole (0.51 g, 3.0 mmol) was added later and stirred at 0 °C for 6 h. The resulting mixture was concentrated in vacuo and then subjected to column chromatography using flash silica as a stationary phase. Compound 1 was obtained using hexane and ethyl acetate (6:4 v/v) as mobile phases. Rf: 0.60 (DCM:MeOH, 9: 1, v/v). Yield 35%; M.P.: 160–170 °C, EI-MS m/z 239.1. ^1^H NMR (300 MHz MeOD) δ ppm: 7.9 (s 1H imidazole), 2.4 (s 3H CH_3_), 5.9 (s 1H C=C), 5.5 (s 1H C=C), 3.02 (s 3H CH_3_) 4.7 (t 2H CH_2_), 4.5 (t 2H CH_2_).

For the second step, a co-precipitation method was adopted for the preparation of narrow range MNPs first by using the previously described protocol (Petcharoen and Sirivat 2012). Here, MNPs was further etched with 3-(Trimethoxysilyl)propyl methacrylate (MPTES) through silanization reaction with slight modification (Saif et al. 2015). Briefly, a MNPs (10 mg/mL) suspension was prepared in ethanol and then MPTES was added in such a manner that the ratio between NPs to MPTES remains 1:6 and left with constant stirring at 60 °C for 4 h. The resultant brownish suspension was washed several times with ethanol and freeze dried. Finally, polymerization was adopted with the aim to functionalize compound 1 on MPTES coated MNPs. Typically, MPTES-MNPs (10 mg/mL) dispersion was prepared in anhydrous ACN under Ar atmosphere. Then, compound 1 (1.20 g, 5 mmol) was added to the above dispersion and after being stirred to 10 min, AIBN (1.32 g, 8 mmol) was added to the resulting mixture and refluxed for 15 h under Ar atmosphere at 60 °C. The prepared Met-MNPs underwent successive washing with ACN and were freeze dried. Met-MNPs were further exploited for their drug entrapment potential using a passive drug loading technique. Briefly, Met-MNPs were incubated with various equivalents of AmpB in methanol for 24 h on a shaker at 200 rpm under ambient conditions. The resulting Met-MNPs-Amp was removed by means of a permanent magnet and washed sequentially with water to remove the unloaded drug and stored at 4 °C for further analysis.

### Characterization of Met-MNPs-Amp

#### Size, size distribution and morphology

The average hydrodynamic diameter and polydispersity index (PDI) of vacant and Met-MNPs-Amp were investigated via Zetasizer (Zetasizer Nano ZS90 Malvern Instruments, Malvern, UK). Concisely diluted nanoparticles were cautiously transferred to a transparent plastic cuvette to avoid any bubble formation. The cuvette was then placed in the cell holder of the instrument and analysis was taken at 90° scattering at 25 °C. The medium viscosity and refractive index were constant and kept at 1.0, 1.33 and 80.4 mPa, respectively. Nanoparticles were also characterized for morphology using atomic force microscopy (AFM, Agilent 5500). A drop of the formulation was placed on a mica slide and air dried at ambient temperature and placed under a microscope. The morphology was investigated at non-contact model.

#### Drug entrapment efficiency determination

Entrapment efficiency is described as the amount of drug entrapped into a carrier with respect to the initial amount of drug added (Manatunga et al. 2017). Therefore, the entrapment efficiency was determined by measuring the amount of unloaded drug at 405 nm by UV spectroscopy (Shimadzu 1800 series, Shimadzu Japan). Drug entrapment was investigated inthe following relation.

$$\% {\text{EE}} = {{\left( {{\text{Q}}_{\text{t}} - {\text{Q}}_{\text{p}} } \right)} \mathord{\left/ {\vphantom {{\left( {{\text{Q}}_{\text{t}} - {\text{Q}}_{\text{p}} } \right)} {{\text{Q}}_{\text{t}} }}} \right. \kern-0pt} {{\text{Q}}_{\text{t}} }} \times 100$$

Q_p_: Quantity of free drug, Q_t_: Quantity of drug added,  % EE: Entrapment efficiency of loaded drug in percent.

#### Fourier Transformed Infrared (FT-IR) Spectroscopy

Fourier transformed infrared (FT-IR, IR-470 spectrometer (Shimadzu, Kyoto, Japan)) analysis was performed in order to elucidate the possible drug entrapment and surface functionalization. Small amounts of powdered nanoparticles were mixed with KBr and subjected to a high pressure of 200 Psi to obtain self-supporting disks.

### Biocompatibility studies

#### Hemocompatibility study

Human blood was obtained from healthy individuals in the University of Karachi, Pakistan following relevant guidelines and regulations (Ethics committee approval ICCBS/IEC/LET/015/2018). Ethylenediaminetetraacetic acid (EDTA) stabilized fresh human blood samples (5.0 mL) were added to 10 mL of phosphate-buffered saline (PBS). Then, red blood cells (RBCs) were isolated via centrifugation at 6000 rpm and washed several times with PBS solution. The purified RBCs were further diluted in 50 mL PBS and Triton X was used as the positive control, respectively. Then, 0.2 mL of diluted RBC suspension and 0.8 mL of Met-MNPs solutions in a range of 200–1000 µg/mL were mixed by vortexing. All sample tubes were kept in static condition at room temperature for 3 h. Finally, the mixtures were centrifuged at 12,000 rpm for 10 min, and 1.5 mL of the supernatant of each sample was transferred to a cuvette. The absorbance values of the supernatants at 540 nm were determined by UV–Vis spectrophotometry. The percent hemolytic activity of RBCs was calculated using the following relation.

$$\% {\text{H}}.{\text{A}} = {{{\text{R}}_{\text{s}} } \mathord{\left/ {\vphantom {{{\text{R}}_{\text{s}} } {{\text{R}}_{\text{c}} }}} \right. \kern-0pt} {{\text{R}}_{\text{c}} }} \times 100$$

R_s_: Absorbance of sample, R_c_: Absorbance of positive control, % H.A: Hemolytic activity in percent.

#### In vitro cytotoxicity

The synthesized nano carrier was screened for its cytotoxicity using MTT assay. Human cervical adenocarcinoma cells (HeLa) (ATCC^®^ CCL-2™), were obtained from American Type Culture Collection (ATCC),and cultured in Roswell Park Memorial Institute (RPMI) 1640 medium, supplemented with 10% foetal bovine serum, 1% minimum essential medium amino acids, 1% l-glutamine and 1% antibiotics at 37 °C with 5% CO_2_ (Rajendran et al. 2019). NIH 3T3 cells (ATCC CRL-1658) were purchased from the ATCC and cultured in Dulbecco`s modified eagle`s medium (DMEM) having foetal bovine serum (10%) (Invitrogen, USA) and antibiotics (streptomycin and penicillin—about 50 U/mL).Both cell lines were incubated into well plates with 96 wells and 8 × 10^3^ and 6 × 10^4^ cells/well thickness, individually, in a (200 µL) refined media. After incubation for about 24 h, fresh media was introduced (200 µL) consisting of Met-MNPs at various concentrations from 25 to 100 μg/mL. Cells incubated in media without NPs were used as the negative control and developed for 48 h. 3-(4,5-Dimethylthiazol-2-Yl)-2,5-Diphenyltetrazolium Bromide solution (MTT) in PBS was introduced into each well (20 µL; 5 mg/mL). The unreacted solution was expelled after 4 h incubation. The resulting formazan crystals were dissolved and introduced to 200 μL DMSO per well before being analysed at 570 nm in a microplate reader. For the positive control and reference, standard Doxorubicin and Cyclohexylamine were used. The following relation was utilized to calculate the % cell viability.

$${\text{Cell}}\;{\text{viability}} = {{{\text{A}}_{\text{t}} } \mathord{\left/ {\vphantom {{{\text{A}}_{\text{t}} } {{\text{A}}_{\text{c}} }}} \right. \kern-0pt} {{\text{A}}_{\text{c}} }} \times 100$$

A_t_: Mean of Absorbance value of Test Sample, A_c_: Mean of Absorbance value of Control.

#### Culture of amoebae

*Acanthamoeba castellanii* of T4 genotype (ATCC 50492) was purchased from American Tissue Culture Collection (ATCC). *A. castellanii* was cultured in growth medium consisting of Proteose peptone (0.75% w/v), yeast extract (0.75% w/v), and d-glucose (1.5% w/v) (PYG) (Anwar et al. 2019b).

#### Amoebicidal assays

Amoebicidal assays were performed as previously described (Rajendran et al. 2019). Briefly, 5 × 10^5^ amoebae were incubated with compounds at various concentrations for 24 h at 30 °C. The positive control used was chlorhexidine, respective solvents were used as solvent controls and RPMI-1640 alone was used as the negative control. A haemocytometer and 0.1% trypan blue solution were used to distinguish between live (unstained) and dead (stained) cells, in trypan blue exclusion assays. The number of viable amoebae determined were represented as % cell death for graphical illustration.

#### Cysticidal assays

*Acanthamoeba castellanii* cysts were prepared by methods described previously (Anwar et al. 2019c). After the formation of mature cysts, 5 × 10^5^ cysts were treated with various concentrations of Met-MNPs-Amp in 24-well plates in the presence of RPMI-1640 and incubated for 72 h at 30 °C. Chlorhexidine was used against *A. castellanii* as the positive control. RPMI-1640 alone was used as the negative control. After the incubation, the remaining viable cysts were enumerated using a hemocytometer by trypan blue exclusion method.

## Results

**Fig. 2:**
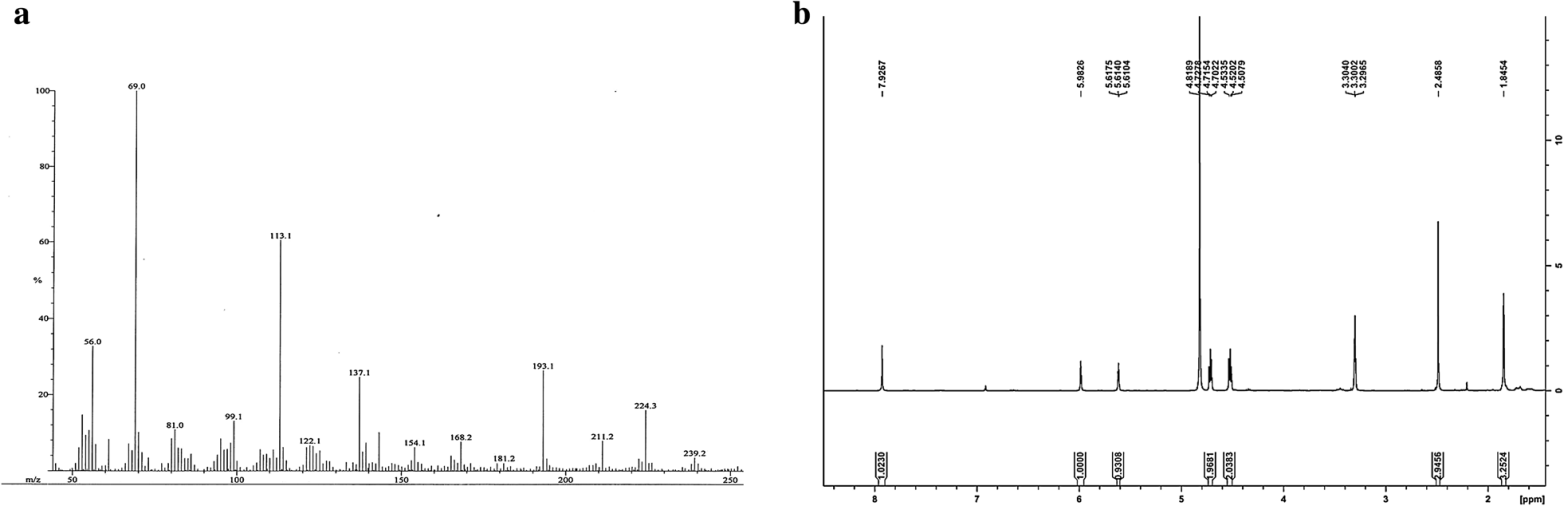
**a** EI-MS spectra of synthesized compound 1, **b**^1^H NMR spectra of synthesized compound 1

**Fig. 3:**
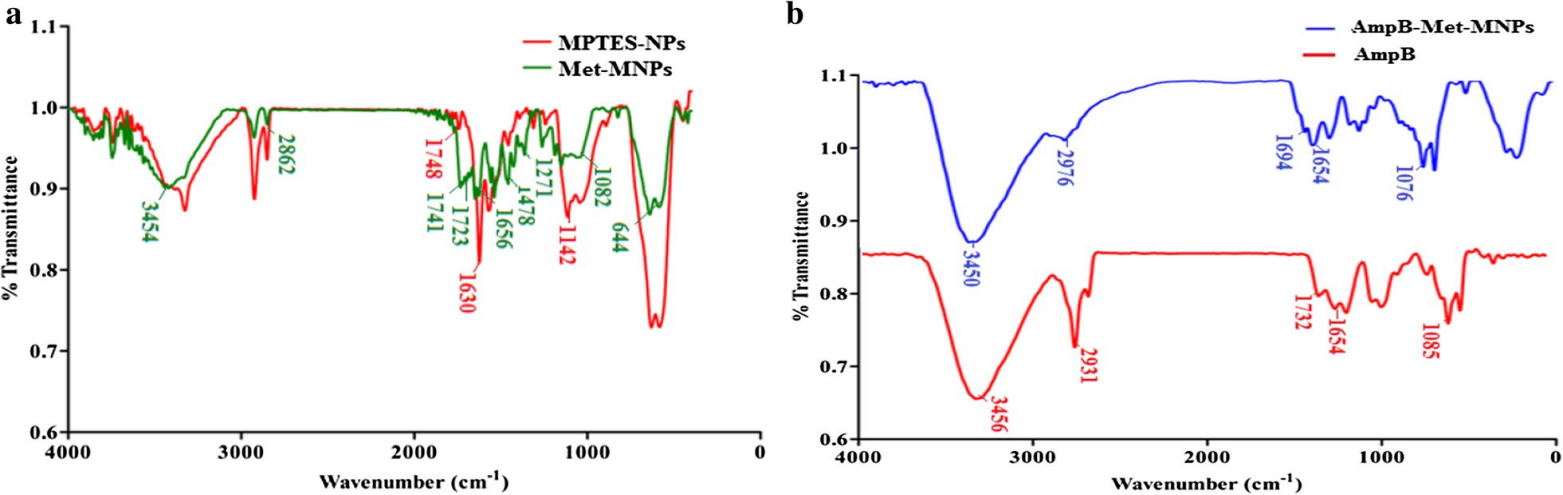
Comparative FT-IR spectra of **a** MPTES-NPs with Met-MNPs. **b** AmpB-Met-MNPs along with AmpB showing the formation of AmpB-Met-MNPs

The synthesis of the compound 1 (2-(2-methyl-5-nitro-1*H*-imidazol-1-yl)ethyl methacrylate) was achieved using carbodiimide coupling reaction (Fig. 1). The EI-MS spectrum showed M^+^ peak at m/z 239.1 which coincides with the theoretical weight of the compound with molecular formula C_10_H_13_N_3_O_4_ (Fig. 2a), respectively. The ^1^H NMR spectra of the synthesized compound 1 shows aromatic singlet around δ 7.9 ppm of 1H (Fig. 2b). Two singlets around δ 2.4 ppm and δ 1.8 ppm of 3H correspond to methyl group. Two singlets at δ 5.9 ppm and 5.6 ppm correspond to olefins. Two triplets at δ 4.7 and 4.5 ppm of 2H corresponds to CH_2_ of metronidazole. The reaction yielded 35% product. FT-IR analysis shows the coating of Met functional moieties onto the surface of MPTES NPs (Fig. 3a) and further AmpB loading (Fig. 3b). The prepared MPTES modified NPs show CH_2_ bands at 2862/cm, for the propyl chain and the presence of (C=O) was associated with the band at 1748/cm. Furthermore, band at 1630/cm corresponds to (C=C) stretch (Ireland et al. 2006; Kawish et al. 2020). Met modified NPs shows two stretching frequencies at 1741/cm and 1728/cm corresponds to two ester (C=O). In addition, symmetric (C=N) aromatic stretch was observed at 1656/cm corresponds to imidazole ring. Moreover, peak at 644/cm on Met-MNPs evidenced that Met is grafted on the surface of MNPs. Amphotericin B reveals characteristic absorption around 1733/cm and 1654/cm corresponding to (C=O) and (C=C) moieties (Jabri et al. 2018). Stretching frequency at 3456/cm corresponds to OH stretching. Amp B-Met-MNPs nanoparticles show slight variation in absorption frequencies -the peak at 1732/cm of carboxylic acid (C=O) was shifted to 1694/cm and the peak at 1654/cm remained unchanged. Frequency at 1085/cm of acetal bond was shifted to 1076 cm^−1^ (Fig. 3b). The absorption at 3456/cm of OH was shifted to 3450/cm which may be attributed to increased chelation of hydroxyl groups with MNPs and secondary hydrogen bonding and $\pi - \pi$ stacking interaction in between drug and the synthesized NPs.

**Fig. 4:**
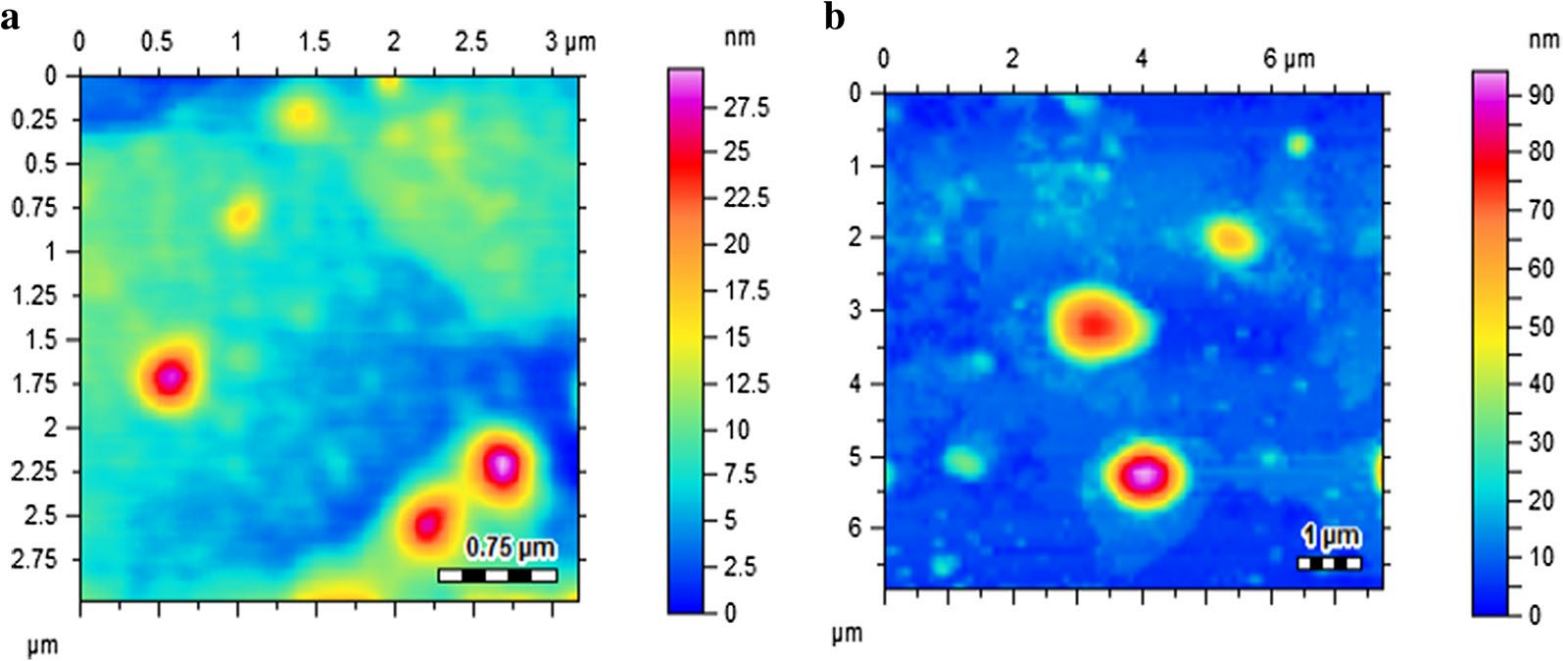
Atomic force microscopic images of **a** Met-MNPs and **b** AmpB-Met-MNPs showing nearly spherical morphology

Particle size and colloidal stability are considered key factors for biological applications (De Jong and Borm 2008). Generally, magnetic nanoparticles are infamous for protein aggregation when introduced in a protein rich media due to their large surface to volume ratio. This undesirable characteristic restricts the biological accumulation of MNPs as biological uptake is mainly dependent upon the colloidal stability of NPs (Oh et al. 2017). The mean hydrodynamic diameter of Met-MNPs and Met-MNPs-Amp was found to be 193.13 ± 6.8 nm and 218.53 ± 13.4 nm (Table 1), respectively. Moreover the PDI values were 0.17 ± 0.05 and 0.26 ± 0.01, respectively. The zeta potential of Met-MNPs and Met-MNPs-Amp was found to be − 13.7 ± 0.9 and − 18.6 ± 4.5 mV (Table 1). The increased size and zeta potential values of Met-MNPs-Amp gives an evidence of drug adsorption on Met-MNPs. Furthermore, aggregation is less likely to occur with high zeta potential due to increased electrostatic repulsion which gives long term stability of our developed NPs. The vacant Met-MNPs nanoparticles were nearly spherical in morphology as revealed by AFM (Fig. 4a). Whereas the amphotericin B loaded Met-MNPs-Amp nanoparticles shows slight distortion in morphology which may be an evidence of successive entrapment of drug onto nanoparticles (Fig. 4b).

**Table 1:** Size, zeta potential, PDI, drug entrapment efficiency at several ratios of Met-MNPs nanoparticles

Nanoparticles	Ratios (drug:NPs)	Size (nm)	PDI	Zeta potential (mV)	Entrapment efficiency (%)
Met-MNPs	None	193.13 ± 6.8	0.17 ± 0.05	− 13.7 ± 0.9	None
AmpB-Met-MNPs	1:1	218.53 ± 13.4	0.26 ± 0.01	− 18.6 ± 4.5	77.05 ± 2.04
AmpB-Met-MNPs	2:1	658.5 ± 510	0.65 ± 0.3	− 17.2 ± 14	68.42 ± 3.21
AmpB-Met-MNPs	3:1	1419 ± 189	1	− 9.43 ± 3.2	52.93 ± 5.32

The characteristic property of any drug carrier is the ability to carry an amount of drug with it, which shows the overall therapeutic outcomes. High therapeutic loading is essential for maintaining prolonged therapeutic effect at the site of action which minimizes the risk of over dosage and its linked toxicities (Imran et al. 2016a, b). Our developed Met-MNPs formulation undergo various blending with AmpB to obtain a formulation containing high amounts of the drug. Interestingly Met-MNPs entrapped 77.05 ± 2.04% of drug at 1:1 ratio excellent colloidal stability as depicted in (Table 1). The higher drug entrapment may be attributed to increased chelation in the form of hydroxyl groups on AmpB, $\pi-\pi$ stacking and hydrogen bonding interactions between drug molecules and Met-MNPs.

**Fig. 5:**
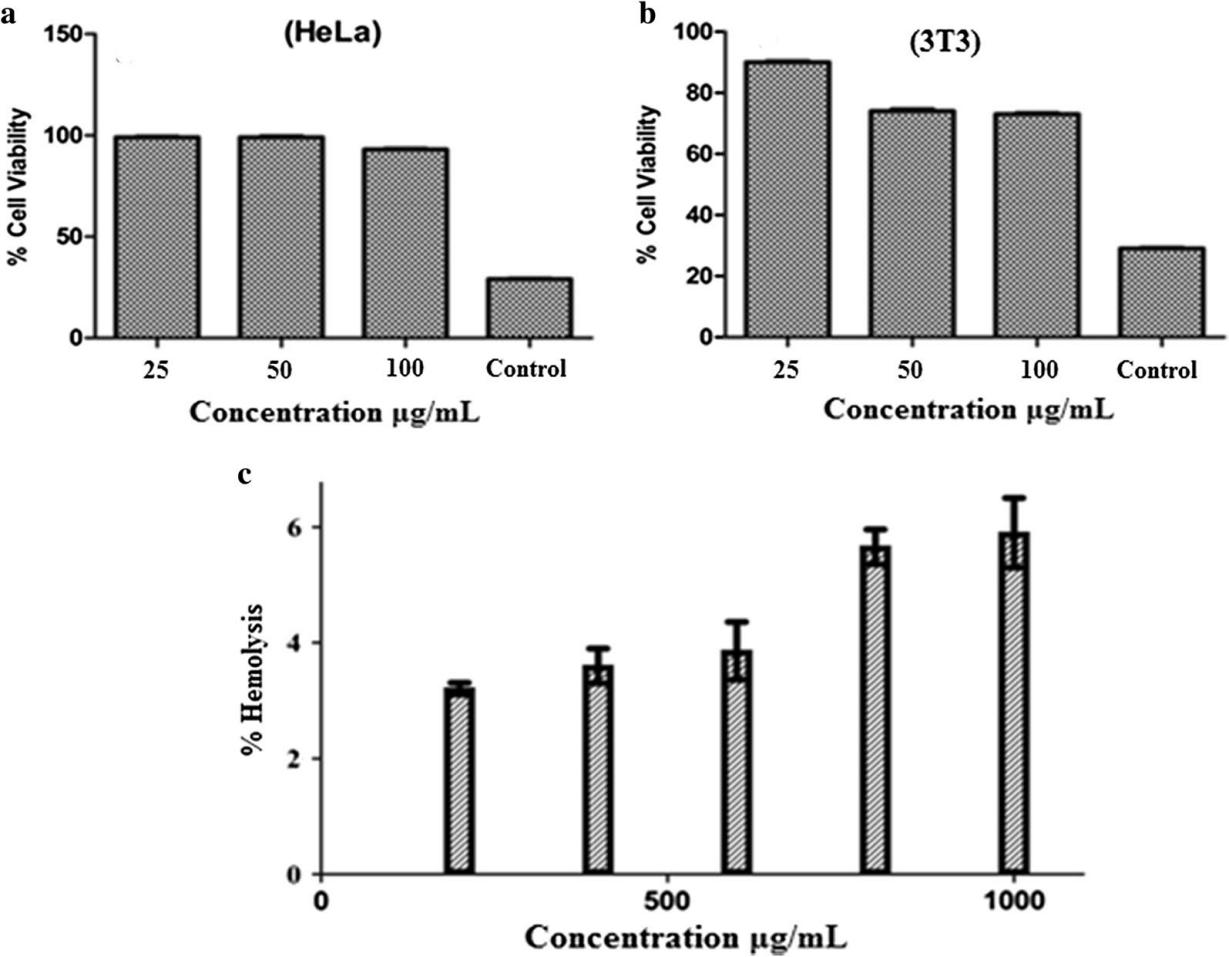
**a** Cell viability profile of Met-MNPs against Hela cell lines. **b** Cell viability profile of developed Met-MNPs against 3T3-NIH cell lines. **c** Concentration dependent hemolytic activity of synthesized Met-MNPs against RBCs

The most predominant factor for any drug delivery excipient is its biocompatibility Therefore, prior to in vivo applications, biocompatibility is considered an essential factor (Fischer et al. 2003). Mouse embryonic fibroblast 3T3 and human cervical cell lines HeLa are commonly used cell lines showing reproducibility. 3T3 and HeLa cell lines were exposed to Met-MNPs-Amp for the evaluation of cytotoxicity using MTT assay. For comparison, doxorubicin and cycloheximide were used as positive controls. Met-MNPs-Amp was incubated at various concentrations against 3T3 and HeLa cell lines. These nanoparticles revealed cell viability in a concentration dependent manner. Experiments conducted against HeLa cell lines, showed that the cell viability was found to be 96 ± 0.65% at concentration of 100 μg/mL, whereas, 3T3 cell lines showed cell viability about 74 ± 1.93% after 48 h as depicted in Fig. 5a, b, respectively. This study reveals that the synthesized nanoparticles are biocompatible. Surface functionalization with biocompatible molecules not only lowers toxicity but enables them the carry drug cargo with it.

The interaction of surface functionalized magnetic NPs with negatively charged membranes have been studied via hemolysis study. The release of hemoglobin from cells determines the membrane destruction characteristics of NPs (Fischer et al. 2003). Titron X-100 was used as 100% values for erythrocytes in this study. Met-MNPs-Amp were used at different concentrations ranging from 200, 400, 600, 800 and 1000 µg/mL and released hemoglobin was quantitatively analyzed at 541 nm (Fig. 5c). Met-MNPs-Amp showed less than 10% hemolytic activity even at a higher concentration of 1 mg/mL indicating the membrane friendly properties (i.e. did not cause any disruption to biological membrane).

**Fig. 6:**
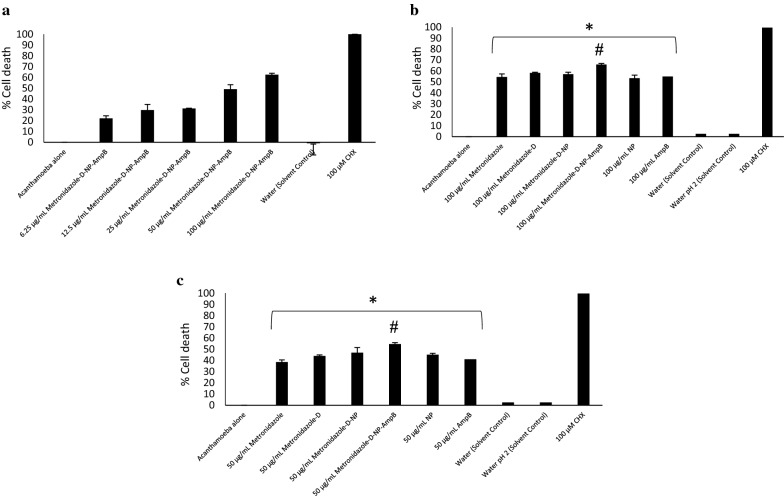
**a** IC_50_ of AmpB-Met-MNPs against trophozoites. **b** Antiamoebic activity against *Acanthamoeba castellanii* trophozoites at 100 µg/mL. **c** Antiamoebic activity against *Acanthamoeba castellanii* trophozoites at 50 µg/mL (*P < 0.05 as compared to negative control)

**Fig. 7:**
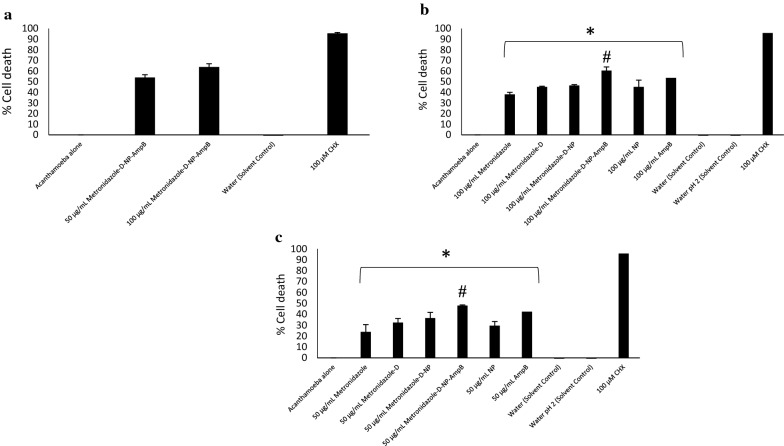
**a** IC_50_ of AmpB-Met-MNPs against cysts. **b** Antiamoebic activity against *Acanthamoeba castellanii* cysts at 100 µg/mL. **c** Antiamoebic activity against *Acanthamoeba castellanii* cysts at 50 µg/mL (^#^P < 0.05 as compared to drugs and nanoparticles alone)

Met-MNPs-Amp demonstrated dose dependent amoebicidal effects from 100 to 6.25 μg/mL (Fig. 6a). These findings suggest that nanoparticles are valuable in developing future treatments against these deadly infections (Anwar et al. 2018). The IC_50_ was found to be 50 μg/mL against *A. castellanii* trophozoites. Met-MNPs-Amp showed significantly improved amoebicidal effects as compared to all respective controls at both 100 and 50 μg/mL (Fig. 6b, c). Met-MNPs-Amp elicited 66% and 55% *A. castellanii* trophozoite death at 100 and 50 μg/mL, respectively. The IC_50_ against *A. castellanii* cysts was found to be 50 μg/mL (Fig. 7a). Met-MNPs-Amp caused 61% cyst death at 100 μg/mL and 48% cyst death at 50 μg/mL (Fig. 7b, c). A significant improvement in the cysticidal activity of Met-MNPs-Amp was seen when compared to metronidazole alone, Met-NPs, as well as NPs alone at both100 and 50 μg/mL (Fig. 7b, c).

## Discussion

Given that the mortality rate of infection due to brain eating amoebae is over 90%, we tested the aforementioned conjugated drug. Met-MNPs-Amp demonstrated dose dependent amoebicidal effects from 100 to 6.25 μg/mL (Fig. 6a). These findings suggest that nanoparticles are valuable in developing future treatments against these deadly infections (Anwar et al. 2018). These findings are not surprising as metronidazole is a common antiprotozoal drug used against *Giardia* and *Entamoeba* and *Trichomonas* (Freeman et al. 1997). It has also been the part of regimen used to treat cases of primary amoebic meningoencephalitis (PAM) (Gupta et al. 2015; Deetz et al. 2003). Metronidazole is also known to inhibit the nucleic acid synthesis of brain-eating amoebae (Mungroo et al. 2019). However, it has shown limited efficacy against the later stages of infection and has been replaced with other drugs (Deetz et al. 2003). On the other hand, amphotericin B has been the corner stone of therapy against *N. fowleri* and *B. mandrillaris* by targeting ergosterol and disrupting membrane (Grace et al. 2015). Albeit its high associated toxicity at the higher dosage required to kill amoebae, the administration of this macrolide is an issue which has been addressed by its incorporation with nanomaterials. Amphotericin B conjugated with gold and silver nanoparticles have shown enhanced antiamoebic effects against *A. castellanii* and *N. fowleri* (Anwar et al. 2019b; Rajendran et al. 2017). In another report, Lemke et al. reported the increased drug delivery of amphotericin B in the form of nanosuspension to the brain against *Balamuthia* infection (Lemke et al. 2010). Since metronidazole, and amphotericin B have been successfully used clinically against brain-eating amoebae infections, we successfully formulated novel drug delivery nanoparticles containing both of these drugs. For this purpose, iron oxide nanoparticles were used as carriers for the first time against these amoebae. Magnetic iron oxide nanoparticles were chemically conjugated with metronidazole which were further loaded with amphotericin B to exhibit synergistic effects. The small size of these nanoparticles ensures the targeted delivery of metronidazole and amphotericin B to their respective active sites in brain. These nanoparticles are also expected to increase the sensitivity of MRI for the detection of amoebae in the brain. Furthermore, the magnetic nature of these nanoparticles can be utilized to isolate amoebae from biological and environmental samples which can be useful platforms for biomedical applications.

In summary, metronidazole conjugated iron oxide nanoparticles loaded with amphotericin B were synthesized. These nanoparticles were thoroughly characterized by FT-IR, AFM, and DLS analyses. These nanoparticles were found to be biocompatible when tested against human and rat cell lines, and also did not show haemolytic activity in vitro. These nanoparticles were designed to exhibit potent antiamoebic effects against the pathogenic parasite *A. castellanii*. This study showcased the antiamoebic activity of magnetic iron oxide nanoparticles for the first time against free-living amoebae. The results from amoebicidal and cysticidal studies against *A. castellanii* showed that these nanoparticles hold potential for future mode of action and in vivo studies.

## Data Availability

Data will be provided upon request on a case to case basis.
